# Neutrophil extracellular traps formation is associated with impaired clot retraction and fibrinolysis in asthma

**DOI:** 10.3389/fimmu.2026.1700840

**Published:** 2026-04-17

**Authors:** Tomasz Misztal, Maria Magdalena Tomasiak-Łozowska, Aleksandra Poznalska, Wiktoria Ciochanowska, Tomasz Rusak, Marcin Moniuszko

**Affiliations:** 1 Department of Physical Chemistry, Medical University of Białystok, Białystok, Poland; 2 Department of Allergy and Internal Diseases, Medical University of Białystok, Białystok, Poland; 3 Student Science Club in the Department of Allergy and Internal Diseases, Medical University of Białystok, Białystok, Poland

**Keywords:** asthma, clot retraction, fibrinolysis, histone H3, myeloperoxidase, neutrophil extracellular traps, neutrophilic asthma, thromboembolism

## Abstract

**Background:**

Asthma is associated with an elevated risk of venous thromboembolism and pulmonary embolism; its etiology is not completely understood.

**Objective:**

This study was designed to investigate whether the formation of neutrophil extracellular traps (NET) is associated with impaired clot retraction and fibrinolysis in the blood of patients with asthma.

**Methods:**

Groups of 50 patients with asthma and 40 healthy subjects were enrolled in this study. Clot retraction kinetics were assessed by optical method (using a digital camera); fibrinolysis was recorded by thromboelastometry; NET predictors in plasma or serum—citrullinated histone H3 concentration, myeloperoxidase activity, and cell-free DNA level—were measured by microplate immunoenzymatic, colorimetric, and fluorometric assays, respectively. Spirometry (FEV_1_ parameter), exhaled NO (FENO), fibrinogen, d-dimers, IgE, and eosinophil, neutrophil, and platelet counts were assessed by standard clinical procedures.

**Results:**

We found the elevated NET predictors in the blood of patients with asthma, which notably correlate with significant inhibition of clot retraction and fibrinolysis compared to the control group. FENO was found to be the most closely correlated with impaired fibrinolysis and clot retraction among all clinical tests.

**Conclusion:**

It was concluded that the formation of NET in the blood of patients with asthma is associated with impairment of clot retraction and fibrinolysis, which may contribute to the formation of unstable thrombi. This may partially explain the elevated risk of venous thromboembolism and pulmonary embolism in asthma.

## Graphical Abstract

**Figure:**
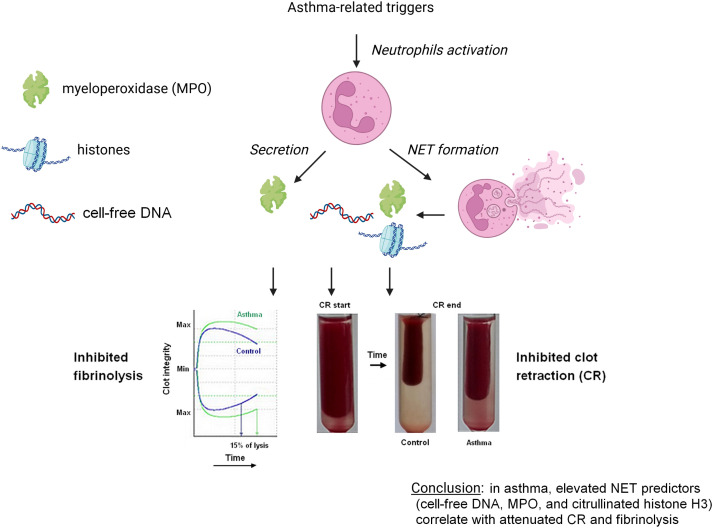

## Highlights

- Asthma is a condition associated with an elevated risk of thromboembolism.
- The formation of neutrophil extracellular traps (NET) in the blood of patients with asthma is notably enhanced compared to healthy subjects, and it correlates with impaired clot retraction and delayed fibrinolysis.
- Attenuation of clot retraction and its lysis may contribute to the formation of unstable thrombi and might partially explain the elevated risk of thromboembolism in asthma.
- Simple laboratory assays of NET predictors in plasma may be useful in the evaluation of thromboembolic risk in patients with asthma.

## 1 Introduction

Bronchial asthma is a condition associated with an elevated risk of venous thromboembolism (VTE) and pulmonary embolism (PE), which has been estimated to be from three- to sevenfold higher compared to healthy subjects (1, 2). The fundamental event leading to PE is the detachment of a thrombus from the vessel wall and subsequent embolization of the pulmonary artery. The mechanism of such detachment comprises mechanical instability of the thrombus due to impaired clot retraction, a process in which activated platelets generate contractile force to efficiently compact the clot structure, making it more mechanically stable, i.e., more resistant to the pressure of flowing blood. Experiments utilizing animal models, in which the attenuation of clot retraction was induced by pharmacological inhibition or by genetic modification of platelets, revealed that efficient contraction of platelet plug or platelet-fibrin thrombus is a primary factor determining their stability under flow conditions (3–5). Correspondingly to PE, thrombotic risk resulting from reduced platelet contractility has been shown to be correlated with the severity and pathogenesis of ischemic stroke (6) and systemic lupus erythematosus (7).

While platelets are the main constituent of arterial thrombi, recent studies indicate that platelet-driven clot retraction is also essential for the stability of venous thrombi, regulating their obstructiveness and embologenicity (4, 7). The extent of contraction has been shown to be the lowest in the tail of venous thrombi, which is the most embologenic part of a venous thrombus (8). There is a close relationship between clot retraction and fibrinolysis, a process of enzymatic digestion of the clot. Clot retraction modulates the rate of fibrinolysis by two mechanisms (1): it promotes internal (physiological) fibrinolysis by elevating local concentrations of both the fibrin-bound fibrinolytic enzymes and their substrate, i.e., contracted fibrin (9, 10), but (2) it also reduces external (pharmacological) fibrinolysis by decreasing clot permeability and the diffusivity of fibrinolytic agents within the clot structure (9, 11). Morphological examinations revealed a high degree of consistency between venous thrombi and pulmonary emboli (12), pointing out that fibrin is the main component of both types of thrombi. Hence, one may suppose that impaired (delayed) fibrinolysis is a factor prolonging thrombus longevity, i.e., the duration of vein occlusion, and elevating the risk of its disengagement by flowing blood. Such an assumption is further supported by numerous observations where altered fibrin structure, toward higher resistance to lysis, correlated with an elevated risk of thrombosis (13–16).

In the blood of patients with asthma, the negative regulators of both processes—clot retraction and fibrinolysis—are likely to occur. These comprise compounds related to the inflammatory response, such as reactive nitrogen and chlorine species, e.g., nitric oxide, peroxynitrite, and hypochlorous acid (HOCl) (17–22). Recently, we demonstrated that HOCl, at physiologically relevant concentrations, may significantly inhibit clot retraction and reduce fibrinolysis in an *in vitro* experimental model (23). The primary source of HOCl in blood is the heme-containing enzyme myeloperoxidase (MPO), which is released into plasma during neutrophil degranulation, a process that is elevated in patients with asthma (24, 25). The second source of MPO in blood is neutrophil extracellular traps (NET), which are web-like structures composed of uncondensed neutrophil chromatin incrusted with histones and other proteins with antibacterial properties, including MPO (26). NET formation has been found to correlate positively with lysis resistance of fibrin clots in experimental models (27, 28).

It has been proposed that in asthma, alterations in small blood vessels (triggered by proteases, bound with allergens, or secreted from activated inflammatory cells) cause platelet activation and further exposure of P-selectin and the release of interleukin-33 (IL-33), belonging to the alarmin class of signaling molecules (29). These phenomena lead to IL-33-mediated infiltration of neutrophils and formation of platelet–neutrophil complexes by direct interaction of platelet-exposed P-selectin and its ligand—PSGL—permanently present on the neutrophil surface. This, in turn, leads to NET formation, enabling pathogen/allergen capture and rapid neutralization (30). Since the lungs are very well perfused and a relatively large organ, it is likely that the existence of a hypercoagulable state may affect hemostasis in systemic circulation through the release of platelet-derived regulators of clotting and fibrinolysis processes, as well as factors initiating NET formation.

While the enhanced formation of NET in the blood of patients with asthma has been reported by different research groups (31–34), the relationship between NET formation and clot retraction and fibrinolysis in bronchial asthma has not been specifically studied. Consequently, this study was designed to examine the connection between inflammation parameters, NET predictors, and parameters describing clot retraction and fibrinolysis in the blood of patients with asthma in comparison with healthy subjects.

## 2 Methods

### 2.1 Ethical considerations

The study protocol was approved by the Ethics of Research Committee of the Medical University of Bialystok (agreement number: APK.002.141.2020). Informed consent was obtained from each patient.

### 2.2 Patient characteristics and inclusion criteria

Fifty asthmatic patients were enrolled in the study as follows: 37 subjects with stable, moderate, inhaled corticosteroid (ICS)-treated allergic asthma (18 women, 19 men; mean age: 46 years; range: 22–67 years) and 13 subjects with severe, unstable, ICS-treated allergic asthma (10 women, three men; mean age: 39 years; range: 19–57 years). Asthma was diagnosed according to the criteria recommended by GINA 2019 (35). Patients with asthma exacerbation, defined below, were excluded from the study. Patients with any other respiratory disease or any concomitant malignant, heart, renal, liver, or collagen disease were also excluded.

Asthma exacerbation was defined as an intensification of clinical symptoms, a decrease in spirometric values, and an increase in the consumption of rescue medications. These changes required intensification of anti-inflammatory treatment (increase in inhaled steroid dose and/or addition of oral corticosteroids) and, if a respiratory tract infection was confirmed, therapy with antibiotics. Infection was ruled out based on the patient’s history and clinical examination, as well as WBC measurement. Patients who had had respiratory tract infections in the month prior to the study were excluded from the study.

Patients with stable, moderate (well-controlled) asthma had been treated with low to medium doses of ICS (250–500 µg of fluticasone propionate or equivalent) at a constant dose for at least 3 months. Stable (well-controlled) asthma was defined as a minimal need for rescue medications (short-acting β_2_-agonists), no exacerbations, and no use of systemic steroids in the previous 12 months. Patients with severe, unstable (poorly controlled) asthma had required one or more hospitalizations for asthma and more than three oral steroid bursts in the previous year. In this group of patients, blood samples were collected at least a month after the last dose of oral steroids to avoid the potential promotion of a hypercoagulable state. However, because of quite frequent asthma exacerbations requiring systemic steroids, the number of blood samples in this group was quite small (*n* = 13), and it was one of the limitations of the study. Patients with severe asthma had been taking high doses of ICS (> 500 µg of fluticasone propionate or equivalent) and long-acting β_2_-agonists for at least 6 months. All patients were atopic and sensitized to common inhaled allergens, as evaluated by skin prick tests.

A total of 40 healthy subjects (24 women, 16 men; mean age: 43 years; range: 31–57 years) participated in the study as a control group. They were free of respiratory tract infection within the 3 months prior to the study and of other significant illnesses known to affect fractional exhaled nitric oxide (FENO) measurements. Patients with asthma and healthy volunteers were nonsmokers who, during the previous year, had not been passive smokers.

Patients and healthy volunteers with a Body Mass Index of more than 30 kg/m^2^ were excluded as a potential confounding factor.

The patients with any diseases associated with hypercoagulable states/thrombotic events, such as previous venous thromboembolism, pulmonary embolism, myocardial infarction, atrial fibrillation, stroke, diabetes, and cancer, were excluded from the study. Moreover, patients taking any medications that interfere with the coagulation system (aspirin and other NSAIDs, ticlopidine, vitamin K antagonists, NOACs, and heparin) were not recruited to the study.

All patients and healthy volunteers were examined by a physician and then underwent FENO measurement and spirometry. Blood samples were collected to determine platelet (PLT), neutrophil (NEU), and eosinophil (EOS) counts, fibrinogen, d-dimers, and serum total IgE.

### 2.3 Blood collection and preparation

Venous blood was collected from healthy volunteers with minimal trauma and stasis via a 21-gauge needle (0.8 mm × 40 mm) into 10 ml polypropylene tubes containing 1 ml of 0.129 M (3.2%) trisodium citrate (S-Monovette, Sarstedt, Nümbrecht, Germany). Platelet-rich plasma (PRP) was obtained by centrifugation of whole blood at 200×g for 20 min. To prepare platelet-poor plasma (PPP), PRP was centrifuged at 2,800×g for 10 min. To prepare serum, serum collection tubes (S-Monovette Serum 7.5 ml, Sarstedt, Nümbrecht, Germany) were used. After blood collection, the tubes were placed upright for 30 min at room temperature, then centrifuged at 1,000×*g* for 10 min at room temperature. Serum layer was collected, and hemolysis was assessed by measuring the absorbance within the Soret band (415 nm), corrected at 380 and 450 nm (36). Samples were stored at − 80°C until further use (within 1 month) for cell-free DNA (cfDNA) measurements. There were no freeze–thaw cycles between serum collection and cfDNA assessment.

### 2.4 Laboratory measurements

Platelet, neutrophil, and eosinophil numbers in whole blood were determined using a Coulter R^©^ Hematology Analyzer (Beckman Coulter Inc., Brea, CA, USA). Serum total IgE concentrations were measured using ImmunoCAP™ Technology (Pharmacia Diagnostics, Uppsala, Sweden). Measurements of FENO and the spirometric index (forced expiratory volume in 1 s [FEV_1_]) were performed as described previously (37). d-Dimer levels were measured with an immunoturbidimetric assay (Innovance d-Dimer; Siemens Healthcare Diagnostics Siemens Healthineers, Erlangen, Germany). Fibrinogen was measured by a standard analytical method.

### 2.5 NET predictor measurements

The MPO activity in plasma samples was assessed using a colorimetric microplate assay (Cat. No. MAK563, Merck, Darmstadt, Germany) according to the manufacturer’s protocol. The plasma concentration of citrullinated histone H3 was measured by an enzyme-linked immunosorbent assay according to the manufacturer’s protocol (Cat. No. 501620, Cayman Chemical, Ann Arbor, Michigan, USA). The cell-free DNA content of the serum samples was quantified using extracellular double-strand DNA-sensitive fluorescent staining as follows: final concentration of 1 μM of Sytox Green (Cat. No. S7020, Thermo Fisher, Waltham, Massachusetts, USA) was added to the diluted (1:1 with sterile saline) serum samples and incubated for 5 min at ambient temperature in the dark. The monochromators of the fluorescent plate reader were set at 504 nm (for excitation) and 523 nm (for emission), respectively (38). Measurements were performed using the TECAN Infinite M Plex 200 PRO multiplate reader (Tecan Group Ltd., Männedorf, Switzerland). DNA concentration was quantified using a calibration curve (prepared with *Saccharomyces cerevisiae* genomic DNA, Cat. No. 69240-M, Sigma-Aldrich, St. Louis, Missouri, USA) covering the DNA concentration range of 0.4 to 140 ng/ml.

### 2.6 Clot retraction recordings

Clot retraction kinetics and the final degree of retraction were measured in recalcified whole blood samples by the optical method described in detail in (18). All measurements were conducted at 37 °C.

### 2.7 Thromboelastometric measurements

Maximum clot firmness (MCF) was evaluated by rotational thromboelastometry (ROTEM Delta, Werfen, Barcelona, Spain). Fibrinolysis was recorded by rotational thromboelastometry and expressed as the lysis onset time (LOT; time to 15% reduction of MCF) parameter, a parameter found to have a better resolution than the maximum lysis parameter (39). Clotting and subsequent fibrinolysis were measured by mixing 320 µl of sodium citrate–anticoagulated whole blood with 20 µl of an activating mixture composed of recombinant tissue factor (TF; Dade™ Innovin™ reagent, Cat. No. 10873566, Siemens Healthineers, Erlangen, Germany) and recombinant tissue plasminogen activator (rtPa; Actilyse^®^, Boehringer Ingelheim, Ingelheim, Germany) in 0.2 M calcium chloride (CaCl_2_) solution. Final concentrations of reagents in the analyzed samples were 75 ng/ml (TF), 125 ng/ml (rtPA), and 11.7 mM CaCl_2_, respectively. All measurements were conducted at 37 °C.

### 2.8 Statistical analysis

Data are presented as median and range (first–third quartiles). Comparisons between groups were performed using the Mann–Whitney U test. Correlations between parameters were assessed using a nonparametric test (Spearman’s rank correlation coefficient [r]). A *p*-value < 0.05 was considered to be statistically significant. Post-hoc power calculations were performed to determine the statistical power of the comparisons between moderate and severe asthma groups.

### 2.9 Artwork

The graphical abstract was created in BioRender (Misztal 2026, https://BioRender.com/t8i47w9).

## 3 Results

The characteristics of the patients with asthma and the healthy controls are shown in **Table 1**. Patients with asthma and control subjects were well-matched for demographic variables.

**Table 1:** Characteristics of healthy control subjects and patients with asthma.

Parameter	Healthy subjects (*n* = 40)	Patients with asthma (*n* = 50)	*p*-value
Age (years)	43 (31–57)	44 (19–67)	0.588
Sex (M/F) (% male)	16/40 (41)	22/50 (44)	0.791
FEV_1_ (%)	101.6 (94.6–104.2)	76.4 (59.5–86.5)	< 0.001
FENO (ppb)	15.5 (8.6–32.2)	65.1 (33.2–83.1)	< 0.0001
IgE (IU/ml)	55.4 (33.2–125.3)	250.4 (75–330.6)	< 0.0001
Neutrophils (cells/µl)	3,100 (1,790–4,640)	4,830 (3,320–7,165)	< 0.001
EOS (cells/µl)	115 (55–160)	210 (100–310)	< 0.05
PLT (10^3^ cells/µl)	272 (221–295)	281 (230–307)	0.162
Fibrinogen (mg/dl)	316 (271–376)	323 (262–415)	0.187
d-Dimers (µg/ml)	0.31 (0.24–0.41)	0.37 (0.31–0.5)	0.209
Clot retraction (CR)			
CR rate (% × min^−1^)	1.55 (1.21–1.8)	1.05 (0.83–1.33)	< 0.0001
Final CR (%)	69 (62–72.5)	59 (54–64)	< 0.0001
Thromboelastometry			
MCF (mm)	60 (56–63)	64 (59–68)	0.0063
Lysis onset time (s)	3,012 (2,719–3,363)	4,312 (3,574–4,884)	< 0.0001
NET predictors			
cfDNA (ng/ml)	7.6 (6.15–10.5)	16.45 (13.45–18.58)	< 0.0001
MPO activity (mU/ml)	11.34 (7.33–16.71)	39.33 (20.79–48.02)	< 0.0001
Citrullinated H3 (ng/ml)	10.94 (7.69–17.23)	24.36 (18.5–32.6)	< 0.0001

Results are presented as median (first–third quartiles).

cfDNA, cell-free DNA; CR, clot retraction; EOS, eosinophils; FENO, exhaled nitric oxide; FEV_1_, forced exhaled volume in 1 s; MCF, maximum clot firmness; MPO, myeloperoxidase; NET, neutrophil extracellular traps; PLT, platelet.

### 3.1 Asthma predictors in patients with asthma and healthy subjects

In comparison with healthy controls, patients with asthma demonstrated decreased FEV_1_ (76% of control), higher FENO (420% of control), and total IgE (452% of control; **Table 1**). Neutrophils were notably higher in count than in healthy controls (156% of control), as well as eosinophils (183% of control), but not platelets (103%; **Table 1**).

### 3.2 Inhibition of clot retraction rate and fibrinolysis, and elevation of clot stability and NET predictors in patients with asthma

**Figure 1:**
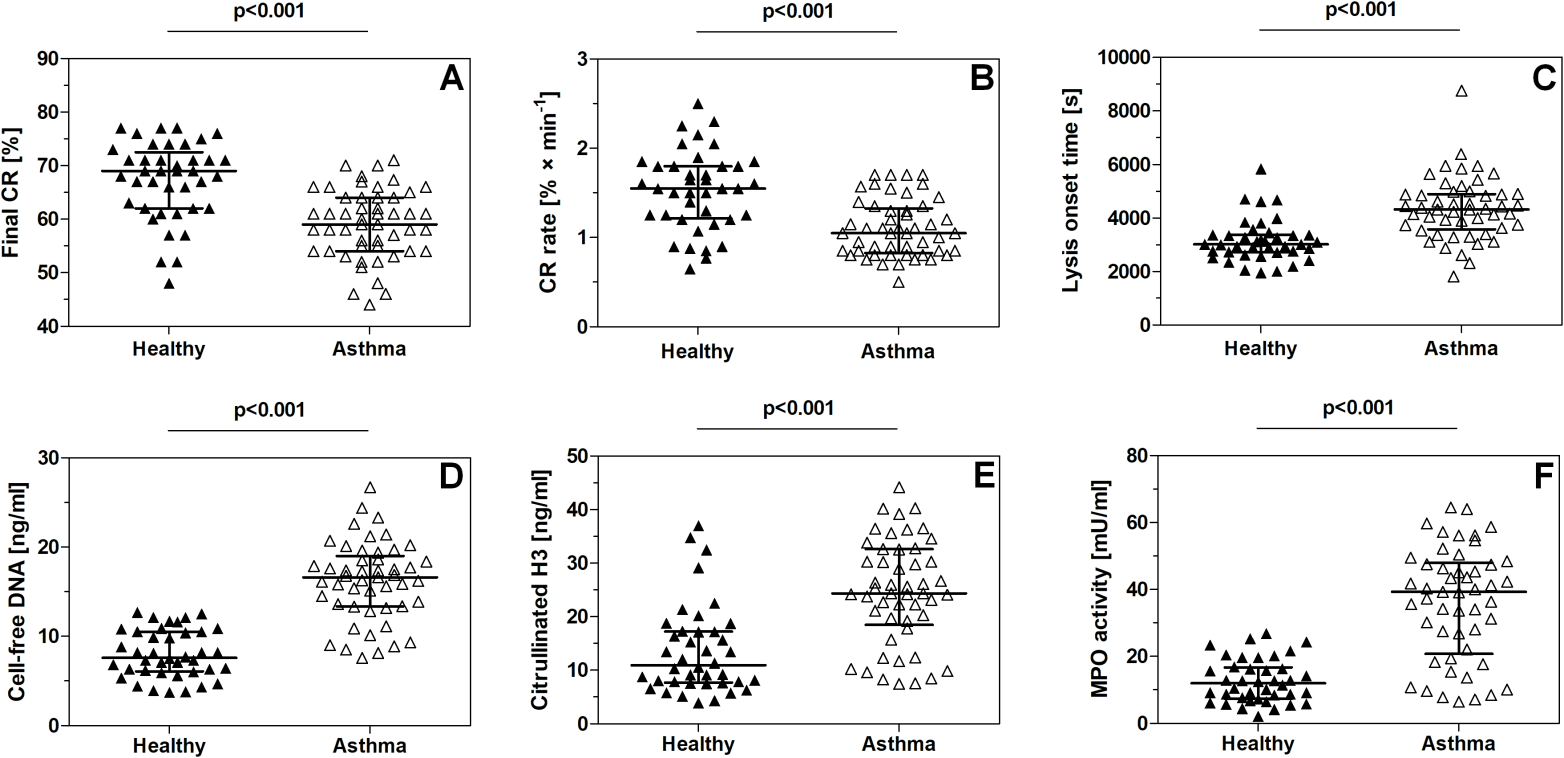
Comparison of parameters describing clot retraction, fibrinolysis, maximum clot firmness, and NET predictors between healthy subjects and patients with asthma. The following parameters were compared: final degree of clot retraction **(A)**, clot retraction rate **(B)**, lysis onset time **(C)**, cell-free DNA level in serum **(D)**, citrullinated histone H3 concentration in plasma **(E)**, and myeloperoxidase activity in plasma **(F)**.

The final degree of retracted clots in the asthma group was approximately 85% of that measured in the control group (**Table 1**; **Figure 1A**), whereas the clot retraction rate (CRR) was significantly lower in the group of patients with asthma (68% of control; **Table 1**; **Figure 1B**). LOT, the parameter reflecting the initial rate of fibrinolysis, was prolonged in the asthma group (143%) compared to the control, reflecting delayed lysis of clots (**Table 1**; **Figure 1C**). A distinct increase (106% of control) in MCF (the parameter characterizing the mechanical stability of the clot) was recorded in the asthma group compared to the control group (**Table 1**). The concentration of cell-free DNA in serum was markedly elevated in the asthma group (216% of control; **Table 1**; **Figure 1D**). Citrullinated H3 histone (citH3) concentration in plasma was elevated in the asthma group (222% of control; **Table 1**; **Figure 1E**), as was MPO activity in plasma (347% of control; **Table 1**; **Figure 1F**).

### 3.3 Correlations between clot retraction rate, fibrinolysis, and NET predictor levels in the control group

In the control group, stronger correlations (compared to patients with asthma) were observed between CR rate and final CR (r = − 0.415, p < 0.01) and between CR rate and MCF (r = − 0.518, p < 0.001). A strong, but slightly weaker compared to the asthma group, correlation was noted between LOT and NET predictors: cell-free DNA (r = 0.789, p < 0.001), citH3 level (r = 0.797, p < 0.001), and plasma MPO activity (r = 0.72, p < 0.001). In the control group, MCF correlated moderately positively with cell-free DNA (r = 0.685, p < 0.001), citH3 (r = 0.588, p < 0.001), and plasma MPO activity (r = 0.626, p < 0.001), and these correlations were stronger compared to the asthma group (all results are presented in **Table 2**).

**Table 2:** Correlations between measured parameters in patients with asthma and healthy subjects.

Variable	citH3	cfDNA	MPO act.	CRR	Final CR	MCF	NEU	EOS	FEV_1_	FENO
Asthma group										
CRR	− 0.599^***^	− 0.626 ^***^	− 0.631^***^	–	0.171	− 0.471^***^	− 0.108	0.088	− 0.074	− 0.305^*^
Final CR	− 0.137	− 0.146	− 0.246	0.171	–	0.018	− 0.022	0.046	0.027	− 0.124
LOT	0.843^***^	0.744^***^	0.918^***^	− 0.606^***^	− 0.093	0.423^**^	0.011	− 0.116	− 0.208	0.517^***^
MCF	0.293^*^	0.316^*^	0.369^**^	− 0.471^***^	0.018	–	0.006	0.055	0.109	0.141
Healthy subjects										
CRR	− 0386^*^	− 0.598^**^	− 0.461^**^	–	0.415^**^	− 0.518^***^	− 0.146	0.121	− 0.093	− 0.145
Final CR	− 0.181	− 0.185	− 0.268	0.415^**^	–	0.149	− 0.077	0.096	0.075	− 0.132
LOT	0.797^***^	0.789^***^	0.720^***^	− 0.382^*^	− 0.180	0.482^**^	0.068	− 0.094	− 0.152	0.309^*^
MCF	0.588^***^	0.685^***^	0.626^***^	− 0.518^***^	0.149	–	0.073	0.023	0.065	0.093

cfDNA, cell-free DNA; citH3, citrullinated histone H3; CR, clot retraction; CRR, clot retraction rate; EOS, eosinophils; FEV_1_, forced exhaled volume in 1 s; FENO, exhaled nitric oxide; MCF, maximum clot firmness; MPO act., myeloperoxidase activity; LOT, lysis onset time.

^*^p < 0.05; ^**^p < 0.01; ^***^p < 0.001—statistical significance.

### 3.4 Correlations between clot retraction rate and NET predictor levels in the asthma group

**Figure 2:**
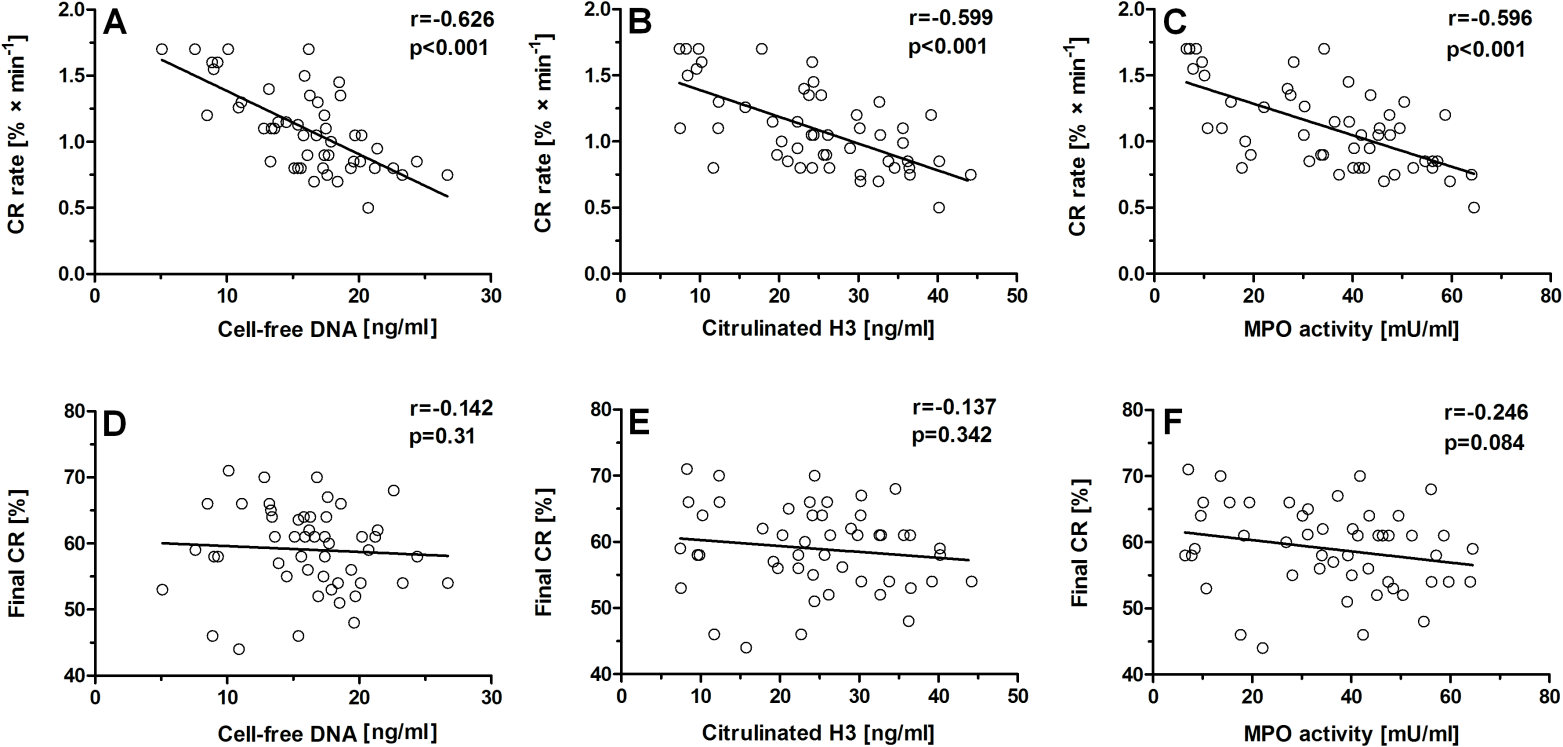
Correlations between parameters describing clot retraction and NET predictors in patients with asthma. The following correlations were established: clot retraction rate vs cell-free DNA level in serum **(A)**, clot retraction rate vs citrullinated histone H3 concentration in plasma **(B)**, clot retraction rate vs myeloperoxidase activity in plasma **(C)**, final degree of clot retraction vs cell-free DNA level in serum **(D)**, final degree of clot retraction vs citrullinated histone H3 concentration in plasma **(E)**, final degree of clot retraction vs myeloperoxidase activity in plasma **(F)**.

In patients with asthma, CR rate inversely correlated with cell-free DNA (r = − 0.626, p < 0.001), citH3 (r = − 0.599, p < 0.001), MPO activity (r = − 0.596, p < 0.001; **Table 2**; **Figures 2A–C**), as well as with MCF (r = − 0.471, p < 0.001; **Table 2**). In the asthma group, a weak negative correlation between the final degree of clot retraction and cell-free DNA (r = − 0.146, p = 0.31), citH3 concentration (r = − 0.137, p = 0.342), and plasma MPO activity (r = − 0.246, p = 0.084; **Table 2**; **Figures 2D–F**) was observed.

### 3.5 Correlations between fibrinolysis, NET predictor levels, and clot retraction rate in the asthma group

**Figure 3:**
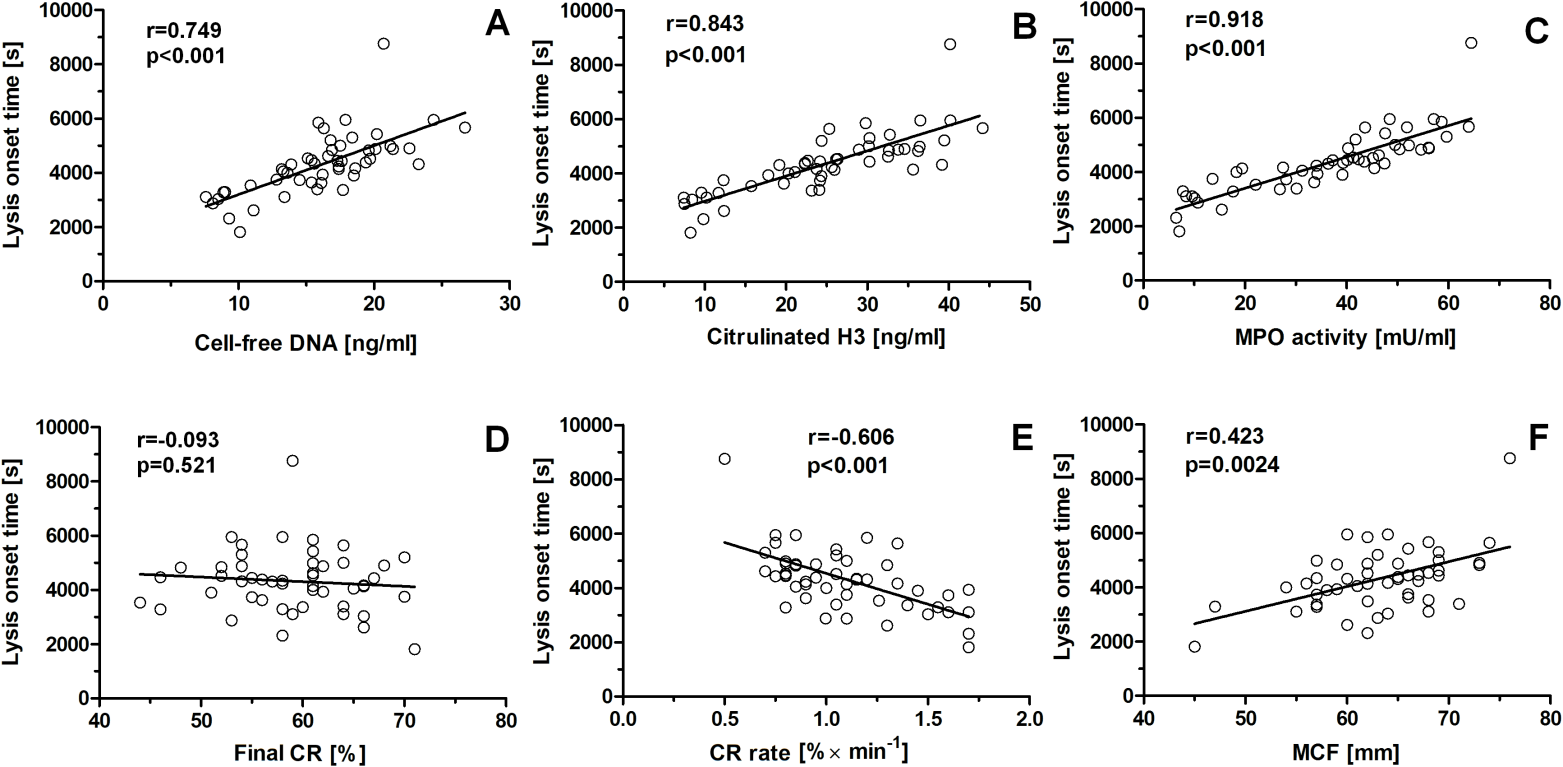
Correlations between lysis onset time and NET predictors, clot retraction rate, and maximum clot firmness in patients with asthma. The following correlations were established: lysis onset time vs cell-free DNA level in serum **(A)**, lysis onset time vs citrullinated histone H3 concentration in plasma **(B)**, lysis onset time vs myeloperoxidase activity in plasma **(C)**, lysis onset time vs final degree of clot retraction **(D)**, lysis onset time vs clot retraction rate **(E)**, lysis onset time vs maximal clot firmness **(F)**.

There were strong positive correlations between LOT and cell-free DNA level (r = 0.749, p < 0.001), citH3 level (r = 0.843, p < 0.001), and plasma MPO activity (r = 0.918, p < 0.001) in the asthma group (**Table 2**; **Figures 3A–C**). No significant correlation between LOT and the final degree of CR was observed (r = − 0.093, p = 0.531; **Table 2**; **Figure 3D**). LOT correlated negatively with CR rate (r = − 0.606, p < 0.001; **Table 2**; **Figure 3E**) and positively with MCF (r = 0.423, p = 0.0024; **Table 2**; **Figure 3F**). MCF was weakly positively correlated with cell-free DNA level (r = 0.316, p < 0.05), citH3 level (r = 0.293, p < 0.05), and MPO activity (r = 0.369, p < 0.01; **Table 2**).

### 3.6 Correlations between clot retraction rate, fibrinolysis, and asthma predictors

**Figure 4:**
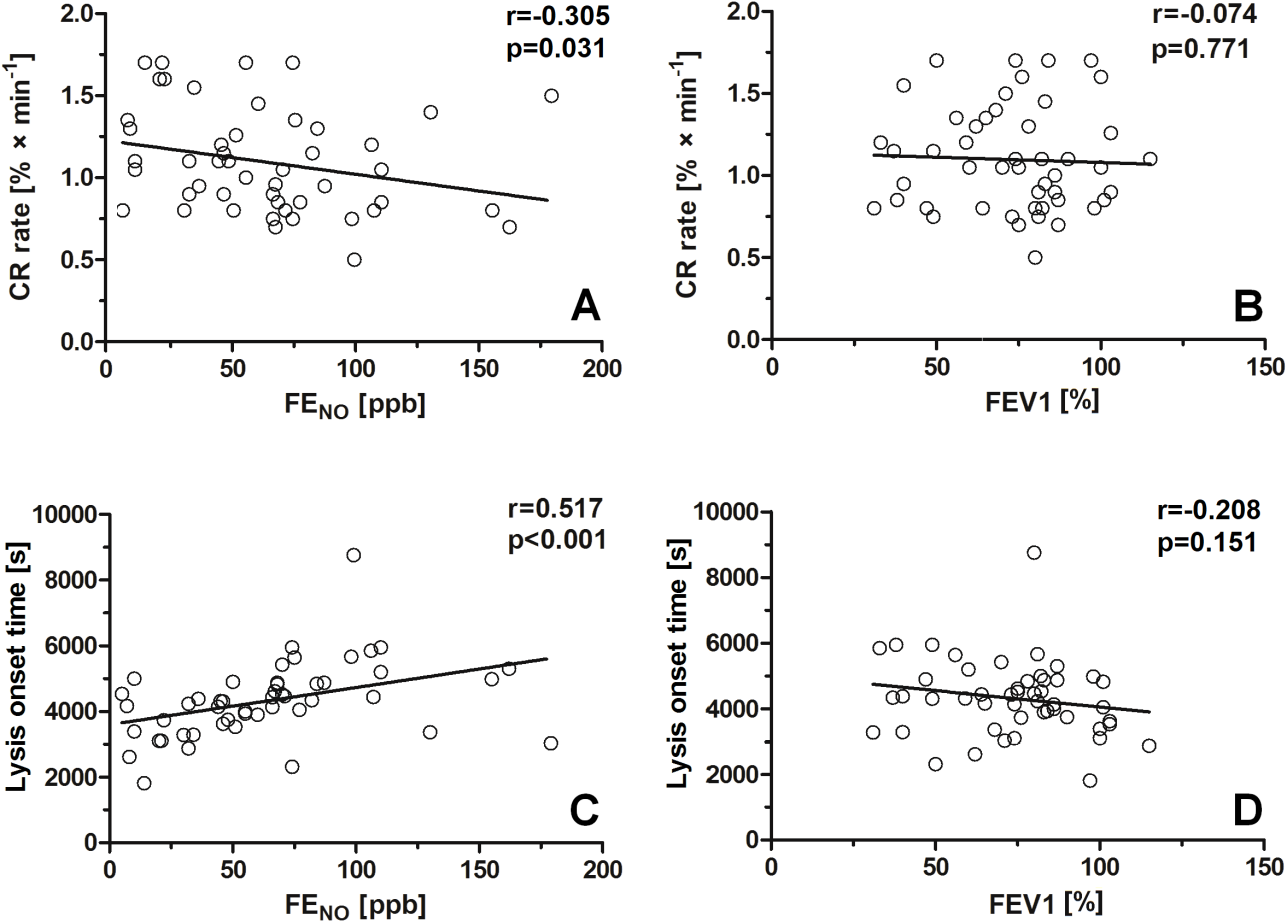
Correlations between clot retraction rate, lysis onset time, and asthma predictors in a group of patients with asthma. The following correlations were established: clot retraction rate vs FENO **(A)**, clot retraction rate vs FEV1 **(B)**, lysis onset time vs FENO **(C)**, lysis onset time vs FEV1 **(D)**.

In the asthma group, CRR was negatively correlated with FENO (r = − 0.305, p = 0.031; **Table 2**; **Figure 4A**), whereas there was no correlation between CRR and FEV_1_ (r = − 0.074, p = 0.771; **Table 2**; **Figure 4B**). In this group, LOT was positively correlated with FENO (r = 0.517, p < 0.001; **Table 2**; **Figure 4C**) and negatively correlated with FEV_1_ (r = − 0.208, p = 0.151; **Table 2**; **Figure 4D**).

### 3.7 Correlations between leukocyte count and asthma predictors with NET predictor levels in the asthma group

In patients with asthma, neutrophil count correlated positively with cell-free DNA level (r = 0.334, p < 0.05), citH3 level (r = 0.298, p < 0.05; **Table 3**), and plasma MPO activity (r = 0.31, p < 0.05). In this group, FENO was positively correlated with cell-free DNA level (r = 0.369, p < 0.01), plasma citH3 concentration (r = 0.387, p < 0.01; **Table 3**), and plasma MPO activity (r = 0.465, p < 0.01). No significant correlations between FEV_1_ and NET predictors were observed (**Table 3**).

**Table 3:** Correlations between neutrophil and eosinophil counts and asthma predictors with measured parameters in patients with asthma and healthy subjects.

Variable	FENO	FEV_1_	MPO act.	citH3	cfDNA
Asthma group					
NEU	0.150	− 0.409^*^	0.310^*^	0.298^*^	0.334^*^
EOS	0.060	− 0.224	− 0.108	− 0.168	− 0.169
FENO	–	0.139	0.465^**^	0.387^**^	0.369^**^
FEV_1_	0.139	–	− 0.129	− 0.091	− 0.113
IgE	− 0.213	− 0.019	− 0.238	− 0.178	− 0.293^*^
Healthy subjects					
NEU	0.186	− 0.209	0.113	0.154	0.175
EOS	0.096	− 0.160	− 0.098	− 0.145	− 0.121
FENO	–	0.192	0.204	0.314^*^	0.219
FEV_1_	0.175	–	− 0.156	− 0.074	− 0.101
IgE	− 0.135	0.092	− 0.143	0.085	− 0.058

cfDNA, cell-free DNA; citH3, citrullinated histone H3; EOS, eosinophils; FEV_1_, forced exhaled volume in 1 s; FENO, exhaled nitric oxide; IgE, immunoglobulin E; MPO act., myeloperoxidase activity; NEU, neutrophils.

^*^p < 0.05; ^**^p < 0.01; ^***^p < 0.001—statistical significance.

### 3.8 Differences in clot retraction rate, fibrinolysis, and NET predictor levels between the moderate and severe asthma groups

**Figure 5:**
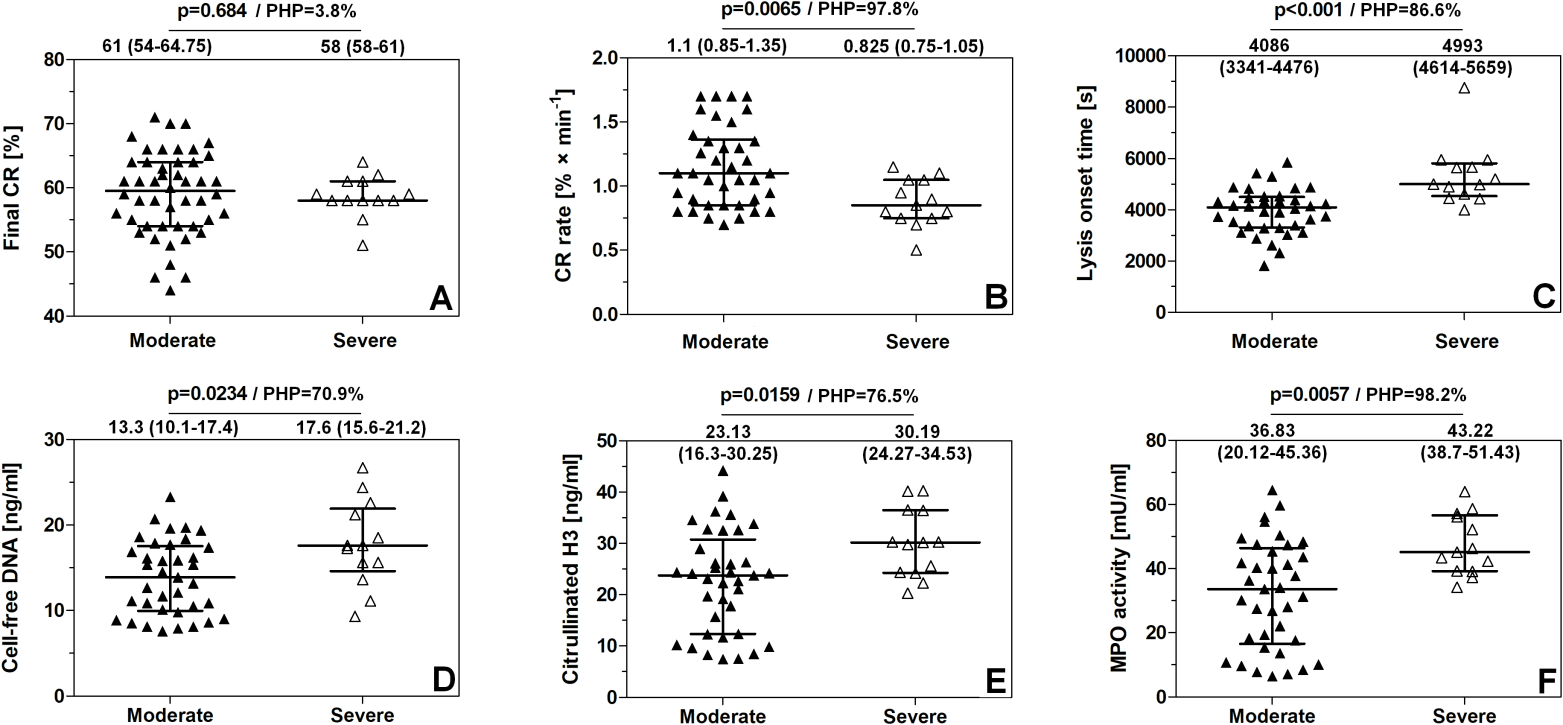
Comparison of parameters describing clot retraction, fibrinolysis, and NET predictor levels between patients with moderate and severe asthma. The following parameters were compared: final degree of clot retraction **(A)**, clot retraction rate **(B)**, lysis onset time **(C)**, cell-free DNA level in serum **(D)**, citrullinated histone H3 concentration in plasma **(E)**, myeloperoxidase activity in plasma **(F)**. Medians and interquartile range (first-third) for each group are presented. PHP, post-hoc power calculations.

There were no statistically significant differences in the final degree of CR between the moderate and severe asthma groups. The CRR was lower by about 25% in the severe asthma group compared with patients with moderate asthma, and LOT was increased in the severe asthma group by about 22% (**Figures 5A–C**). NET predictors, i.e., cell-free DNA, citrullinated H3 histone concentration, and MPO activity, were elevated in the severe asthma group by about 32%, 17%, and 30%, respectively (**Figures 5D–F**). Post-hoc power (PHP) calculations were performed to assess the statistical power of this part of the study, i.e., the probability (expressed as a percentage) of correctly detecting a true difference between the studied groups; a value of 80% is commonly accepted as a minimal conventional standard.

## 4 Discussion

Hereby, we confirm the previously reported inhibition of clot retraction and fibrinolysis in the blood of patients with asthma and introduce the evidence that these phenomena are correlated with NET predictors, which, from the perspective discussed here, may potentially be used as markers supporting the evaluation of hemostatic malfunctions in asthma. Furthermore, based on the obtained results, we conclude that a significant part of the observed hemostatic abnormalities may be linked to the action of MPO-generated HOCl.

In this study, we demonstrate that clot retraction (its rate as well as the final degree of contraction) and fibrinolysis rate (measured as LOT; i.e., time to obtain 15% of clot lysis in thromboelastometric measurement) are significantly reduced compared with healthy subjects (**Figure 1**; **Table 1**). Moreover, the differences between the moderate and severe asthmatic groups were also significant, reflecting the association between the observed hemostatic abnormalities and disease severity (**Figure 5**). Interestingly, a classical test reflecting the degree of ongoing inflammation in lungs, FENO, appeared to be a better predictor of impaired fibrinolysis (r = 0.517, p < 0.001) than of attenuated CRR (r = − 0.305, p < 0.05). It is worth noting that, despite the higher neutrophil count in the blood of patients with asthma, it only slightly correlates with NET predictors (**Table 3**). This suggests that the presence of NET predictors is linked to neutrophil activation status rather than their abundance in the blood. Increased activation of blood neutrophils has been reported in patients with asthma by other authors (25, 40); however, more detailed investigation of the relationship between measurable neutrophil activation markers (e.g., CD11b exposure) and hemostatic abnormalities in patients with asthma is a direction for future studies.

In the asthma group, the reduced CRR was strongly negatively correlated with LOT (r = − 0.606, p < 0.001). It is worth noting that LOT reflects internal fibrinolysis, i.e., a physiological type of fibrinolysis in which all fibrinolytic factors are present within the clot structure. Such a negative correlation between CRR and LOT in asthma is in line with observations by Tutwiler et al. that efficient clot contraction enhanced the rate of internal fibrinolysis (~ 2-fold) by providing closer proximity of the fibrin fibers, i.e., increasing the concentration of substrate for fibrinolytic enzymes (9). Interestingly, such a relationship between CRR and LOT was stronger in patients with asthma than in the control group (r = − 0.606, p < 0.001 vs. r = − 0.382, p < 0.05, respectively), suggesting that the reduced CRR might be one of the main causes of attenuated fibrinolysis in the patient group. Surprisingly, there were no statistically significant correlations between LOT and the final degree of retraction in both groups. One potential explanation of this inconsistency came from the analysis of clot retraction on blood flow in a vessel lumen. According to Litvinov and Weisel, if a thrombus blocks the vessel lumen by 80%, the volumetric blood flow rate will decrease to only 4% of the initial level. On the other hand, if the degree of thrombus contraction increases by only one-tenth of its initial volume, the blood flow will increase 1.6-fold (5). Therefore, this may be interpreted to mean that the final degree of retraction is likely associated with the extend of ischemia after the completion of the thrombus formation process, whereas the propagation of retraction (i.e., CR rate) is more important in the stabilization of the clot (minimizing the risk of detachment of a premature clot by flowing blood) and in providing an adequate fibrinolysis rate.

Concluding, CRR seems to have better resolution value in the prognosis of hemostatic malfunction in asthma than the final degree of CR, which is the basis of a historically—and still currently—performed test for clot retraction in diagnostic laboratories (based on the total volume of extruding serum by a retracting clot) (41).

LOT also correlated moderately (r = 0.423, p < 0.01) with MCF, a thromboelastometric parameter reflecting internal clot integrity, which, *per se*, was distinctly elevated in the group of patients with asthma (p < 0.01). Previously, we showed that MCF is correlated with fibrin clot density (strong correlation, r = 0.860, p < 0.001, please see **Table 2** in (20)). In our other reports, we showed that the *in vitro* exposure of human plasma to physiologically relevant concentrations of HOCl (a main product of MPO) produced altered fibrin clot architecture toward more dense, highly crosslinked, and lysis-resistant structures (23, 42). Here, we show a weak but close to moderate (r = 0.369, p < 0.01) correlation between MCF and MPO activity, which suggests a potential link between the observed elevation of MCF and the production of HOCl in the blood of patients with asthma. It also points to MPO activity as a potential factor modulating not only the CR rate (reported previously by our team (23)) but also the fibrinolysis rate.

To evaluate the correspondence between HOCl production and the observed inhibition of CRR and fibrinolysis, we measured MPO activity in plasma samples collected from patients with asthma and the control group. In parallel, we also measured citrullinated histone H3 levels in plasma and cell-free DNA in serum, which, together with MPO, are considered NET markers/predictors (43). We measured MPO activity, rather than its plasma concentration, due to the possible inhibitory effect of nitric oxide (NO) on MPO catalytic activity (44). In asthma, a constitutive production and release of NO into the bloodstream by endothelial cells coexists with elevated NO synthesis by an inducible isoform of NO synthase (iNOS) released by activated leukocytes (45), as well as with an iNOS-independent mechanism of NO production that relies on the reaction between l-arginine and reactive oxygen species (46). As a net result, MPO functions in an environment replete with NO; therefore, MPO level and its activity in blood may not be linearly dependent on its concentration.

Of importance, correlations between LOT and NET predictors were stronger (r = 0.744, p < 0.001; r = 0.843, p < 0.001 and r = 0.918, p < 0.001 for cell-free DNA, citrullinated H3, and MPO activity, respectively) than correlations between these predictors and the reduced CR rate (− 0.626, p < 0.001; r = − 0.599, p < 0.001; and r = − 0.631, p < 0.001, respectively). Such distinct correlations indicate that elevation of NET-associated markers in the blood of patients with asthma can be a factor worth considering in the context of hemostatic abnormalities in patients with asthma.

There are candidates for the molecular factors engaged in a possible mechanism linking NET with fibrinolysis and clot retraction disturbances. One probable link is the enhancement of thrombin generation observed in the presence of NET constituents such as histones and extracellular DNA, resulting from activation of the intrinsic (factor XII-dependent) pathway of coagulation (by extracellular DNA) and from platelet procoagulant responses triggered by histones, respectively (47). It is well established that fibrin clots formed under high thrombin conditions are denser and more resistant to lysis than the structures formed in the presence of lower thrombin concentrations (48). However, it seems unlikely that this mechanism may coincide with the reduction of CRR since, as we showed previously, retraction propagates faster with the elevation of tissue factor concentration (which translates into amplified generation of thrombin, please see **Figure 3A** in the reference number (49)). On the other hand, HOCl, the main product of MPO in biological fluids, has been identified as a factor that inhibits both processes—clot retraction and fibrinolysis—through different mechanisms. Clot retraction is reduced by this MPO product via diminished energy production in platelet mitochondria, a prerequisite for the generation of contractile force by activated platelets (21). Diversely, fibrinolysis may be impaired by HOCl through its oxidative action on fibrinogen molecules, resulting in an altered pattern of the assembly of such affected protofibrils toward the fibrin structure, which is denser and more resistant to lysis (23, 50). Additionally, the recent study by Komorowicz et al. revealed that fibrinolysis may be directly impaired by core histones (both native and citrullinated), which directly inhibit plasmin via interactions with its kringle domains (51).

Considering elevated MPO activity and citrullinated H3 levels, along with the established functional connection between CR and fibrinolysis—in that efficient contraction of a clot enhances its internal lysis (9)—one may hypothesize that, in the blood of patients with asthma, delayed fibrinolysis may result from two independent phenomena, i.e (1)., impaired CR (likely due to HOCl action on platelets) and (2) HOCl/NET-associated alteration of a fibrin net structure and its antiplasmin (due to the abundant presence of histones).

Presented here adhere to clinical reality in several areas. Neutrophilic inflammation plays a key role in the pathophysiology of many chronic lung diseases. The formation of NET has been suggested as a key mechanism of neutrophilic lung diseases, including asthma, chronic obstructive pulmonary disease (COPD), cystic fibrosis, and even bronchiectasis (52). NETs are large, web-like structures consisting of extruded neutrophilic DNA and antimicrobial proteins that can bind pathogens, prevent microbial dissemination, and degrade bacterial and virulence factors. However, the release of excessive amounts of proteases, antimicrobial proteins, DNA, and histones may also lead to tissue damage, impaired mucociliary clearance, impaired bacterial killing, and increased inflammation. A number of studies have revealed associations between airway NET formation and greater disease severity, increased exacerbations, and overall worse disease outcomes across the spectrum of airway diseases, including asthma (reviewed in (52)).

Pulmonary embolism (PE), a blockage of the lung arteries, is a potentially life-threatening disease (53). At present, there is increasing evidence suggesting that asthma is associated with greater risks of PE (1, 2, 54–58). In the report published by Lee and Fu, the authors conclude that the frequency of pulmonary embolism in patients with asthma may rise with higher disease severity, increased incidences of asthma exacerbation and hospitalization, and the use of inhaled or systemic steroids (52), as was demonstrated previously (1, 2, 54, 57, 59, 60). Consistently, in the study performed by Chung et al., the hazard ratio of PE in asthmatics increased with an increased number of emergency room visits and hospital admissions (27). Of importance, Majoor et al. observed a higher risk of pulmonary embolism in patients with asthma treated in tertiary asthma clinics; moreover, such patients are likely to reflect the most severe cases (1). One may therefore assume that the quality of healthcare and glucocorticoid use are particularly essential factors affecting the overall PE risk in patients with asthma.

Glucocorticoids (GKS), commonly used in asthma treatment, are associated with endothelial dysfunction, hypercoagulability, and stasis (61–63). Systematic reviews by Van Zaane et al. and Majoor et al. revealed that glucocorticoids can decrease fibrinolysis (64, 65). Systemic glucocorticoids are more likely to be used in severe asthma than in less severe asthma, which could also could contribute to the association between asthma severity and VTE risk (54).

In our previous reports, we observed significantly reduced CRR in patients with steroid-naive asthma as well as in those with steroid-treated asthma, compared with healthy controls (18, 20). In the present study, in the group of patients with severe asthma, CRR was lower by about 25% compared with patients with moderate asthma (p < 0.01). The LOT parameter was higher in the severe asthma group by about 22% (p < 0.001). NET predictors—MPO activity, cell-free DNA level, and citrullinated H3 histone concentration—were elevated in the severe asthma group by about 17% (p < 0.01), 32%, and 30% (p < 0.05), respectively. All these differences between the groups of patients with asthma were statistically significant. These patients had required more than three oral steroid bursts in the previous year. This increased consumption of oral GKS could be a potential cause of altered parameters of clot retraction and fibrinolysis. On the other hand, in severe asthma, neutrophilic inflammation can be present, and therefore, increased parameters of NET activation could also be observed.

Neutrophilic inflammation remains a feature of chronic airway diseases (including asthma); therefore, it has proven most challenging to understand and therapeutically target (52). Notably, up to 50% of patients with symptomatic asthma could be classified as neutrophilic (66–68); therefore, currently, there is no direct anti-inflammatory treatment for this group (52).

To sum up, we could suggest that, in our clinical practice, we should give much more serious attention to patients with asthma, especially those with severe disease. This is because, on the one hand, there is a systemic increased risk of PE and DVT, and on the other hand, increased NET predictors and, therefore, an increased risk of neutrophilic (so far difficult to treat) inflammation. The latter is likely to be additionally associated with hemostatic abnormalities, i.e., inhibited clot retraction and fibrinolysis. The role of NET in asthma is still under debate, and NET represent a potential target for the development of new therapeutic strategies (52).

Collectively, the presented study highlights a novel element in our understanding of altered hemostasis in asthma, i.e., a strong correlation between NET predictors (cell-free DNA, citrullinated H3, and MPO activity) and attenuated fibrinolysis, and a moderate correlation of these predictors with inhibited clot retraction. This suggests meaningful resolution of NET predictor levels as a potentially useful tool in the evaluation of prothrombotic blood phenotype in patients with asthma associated with reduced fibrinolysis and clot retraction. We also propose that MPO product—HOCl—is a substantial factor linking the elevation of NET markers with reduced fibrinolysis and clot retraction in the blood of patients with asthma. Considering the low number of patients in the severe asthma group (*n* = 13), the differences between moderate and severe asthma may be under- or overestimated (please see **Figure 5** for *post-hoc* power calculations). Therefore, this part of the study should be considered a pilot, encouraging the conduct of a large-scale study to better determine the clinical significance of the observed differences between asthma groups.

The usefulness of relatively fast and simple microplate methods to assess NET predictors must be emphasized. However, the development and introduction of clinically validated antibodies enabling the measurement of NET markers by flow cytometry is likely to support the diagnostic process even further. Validation of the resolution and, hence, the efficacy of specific tests should therefore be a determinant of future research endeavors.

## Data Availability

The original contributions presented in the study are included in the article/supplementary material. Further inquiries can be directed to the corresponding authors.
